# Supplementary material for: Dynamic Transitions for Fast Joint Acquisition and Reconstruction of CEST‐Rex and T1
Source: Magn Reson Med. 2025 Nov 25;95(4):2153–68. doi: 10.1002/mrm.70198 (PMC12850581; doi:10.1002/mrm.70198)
Supplement: Supplementary file 1 — Document S1. Conventional CEST measurement using a 3D SOS sequence. Document S2. Effect of saturation time Tsat on reconstruction accuracy. Document S3. Test‐Restest experiment for the phantom measurement. Document S4. Further investigation of differences between QUASS MTRAREX and Rex. Figure S1. B0 and B1 field maps of the in‐vivo measurement. Table S1. Optimization and regularization parameters for all experiments. [file MRM-95-2153-s001.pdf]

Document S1 - Conventional CEST measurement using a 3D SOS sequence

This document contains the results of a conventional CEST measurement using a 3D stack-of-stars (SOS) sequence for comparison with the 3D GRE sequence described in the main text. The same readout and saturation parameters as described in the main text were used, but the saturation and 3D SOS readout was applied in the conventional way, i.e. with $T_{rec} = 3.5$ s and without the proposed model-based reconstruction. Instead, for each offset all acquired spokes (128 per partition, 3 partitions) were reconstructed to form a single image. After inverse Fourier transform in z-dimension, reconstruction was performed using BART. The resulting data was normalized against the first acquired offset (-1500ppm) in the same way as for the 3D GRE data. The same reconstruction was also performed for a reduced data set, where only the first 64 spokes were used for each partition.

Figure 1 shows the resulting images of the center slice at 2ppm and the resulting z-spectra. The 3D

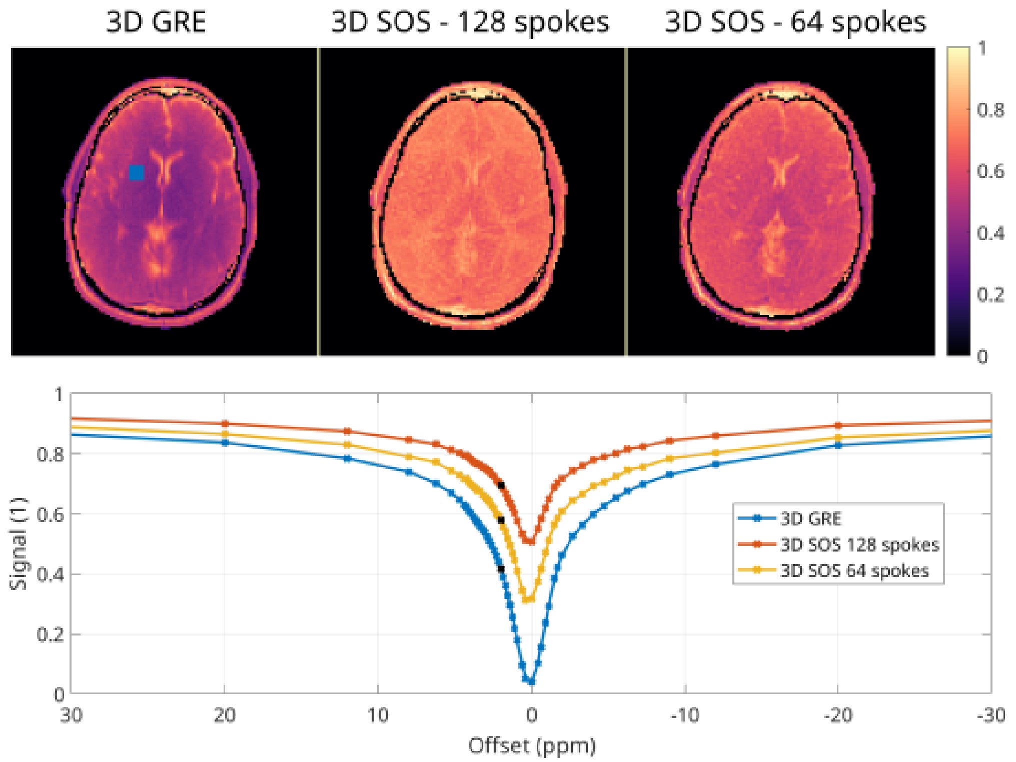

Figure 1: Images at 2ppm and resulting z-spectra from the ROI marked in blue in the 3D GRE image. The offsets of the shown images (2ppm) is marked by the black dot in the spectra

SOS images show a different contrast, which is expected as the 3D SOS sequence repeatably samples the k-space center, thus capturing the T_1 decay during the readout. The same can be seen in the z-spectra, the 3D SOS imaging show higher values over the whole z-spectrum, as the resulting image is the weighted average (depending on the number of spokes) between the starting magnetization after CEST saturation and the steady-state magnetization. This makes the 3D SOS readout unsuitable

for conventional CEST imaging. Only by reducing the number of spokes towards one can a similar contrast to the 3D GRE sequence be achieved, but this leads to a significant decrease in SNR as the undersampling artifacts becomes more pronounced. The 3D SOS sequence can, however, be used very successfully for steady state CEST imaging, as the magnetization is kept constant by the interleaved saturation and readout [1] [2] or in combination with model based reconstruction [3]. The MTR_{AREX} maps calculated from all three z-spectra for Figure 1 are shown in Figure 2. As

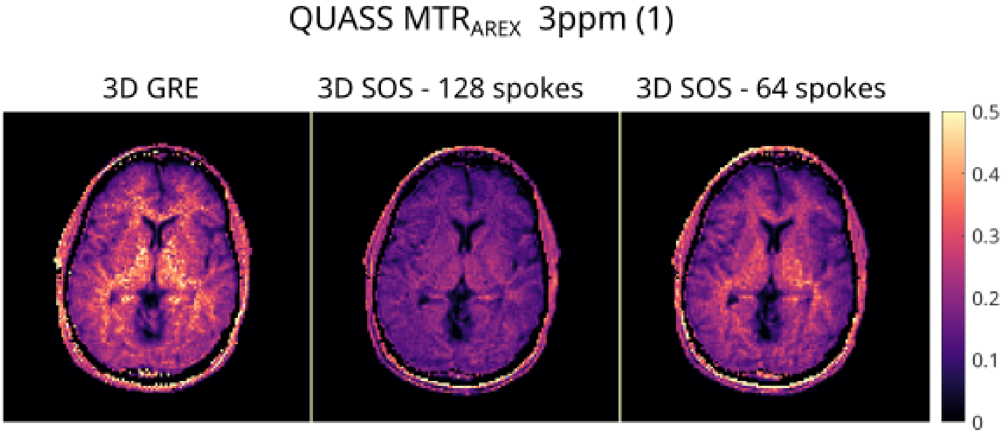

Figure 2: QUASS MTR_{AREX} at 3ppm for 3D GRE, 3D SOS using 128 spokes and 64 spokes.

expected from the spectra, the 3D SOS maps show reduced values compared to the standard 3D GRE sequence.

References

[1] P. Han, K. Cheema, T. Cao, et al., "Free-breathing 3d cest mri of human liver at 3.0 t," Magnetic Resonance in Medicine, vol. 89, pp. 738–745, 2 Feb. 2023, ISSN: 15222594. DOI: 10.1002/MRM.29470.

[2] Q. Tao, Z. Chen, Y. Zhang, W. Zhang, Y. Xu, and Y. Feng, "Renal 4d cest-mri under free breathing," https://doi.org/10.1117/12.3048046, vol. 13270, pp. 99–103, Sep. 2024, ISSN: 1996756X. DOI: 10.1117/12.3048046.

[3] M. Huemer, C. Stilianu, and R. Stollberger, "Transient model based cest imaging—tcest," in Magnetic Resonance Materials in Physics, Biology and Medicine 2024 37:1, vol. 37, Springer, Sep. 2024, pp. 31–33. DOI: 10.1007/S10334-024-01191-6.

Document S2 - Effect of saturation time T_{sat} on reconstruction accuracy

For this experiment, the same phantom and sequences as described in the main text for the phantom measurement experiment were used. Only the saturation time T_{sat} was varied from 1s to 4s. This was done to investigate the effect of saturation time on the reconstruction accuracy. Results are compared to the conventional CEST measurement with a saturation time of 4s.

The sequence durations were 5:00min, 5:30min, 6:00min and 7:55min for the proposed sequence and 9:20min for the conventional CEST method. Reconstruction, pre- and post-processing were done as described in the main text.

The resulting CEST maps are shown in Figure 3. The CEST maps show that the variance of the CEST signal decreases with increasing saturation time, which is expected as the steady-state is reached. Especially noticeable is the decrease in variance in the background of the phantom, where the CEST signal should be zero. Means and standard deviations of the values in the ROIs are shown in Figure 4. And the Bland-Altman plots in Figure 5 show the differences between the different saturation times and the conventional CEST method. The same trend can be observed in Figure 4,

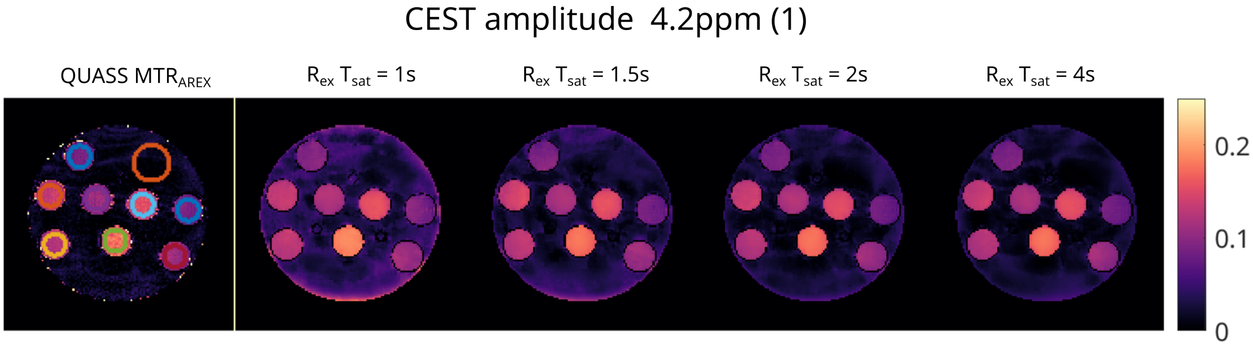

Figure 3: Effect of saturation time on the CEST signal.

and the Bland-Altman plots in Figure 5 show both a decrease in variance and a decrease in the mean deviation from the conventional CEST method with increasing saturation time. From these results, T_{sat}=1.5s was chosen as the optimal saturation time for the phantom measurement experiment, as it provides a good compromise between measurement time and reconstruction accuracy.

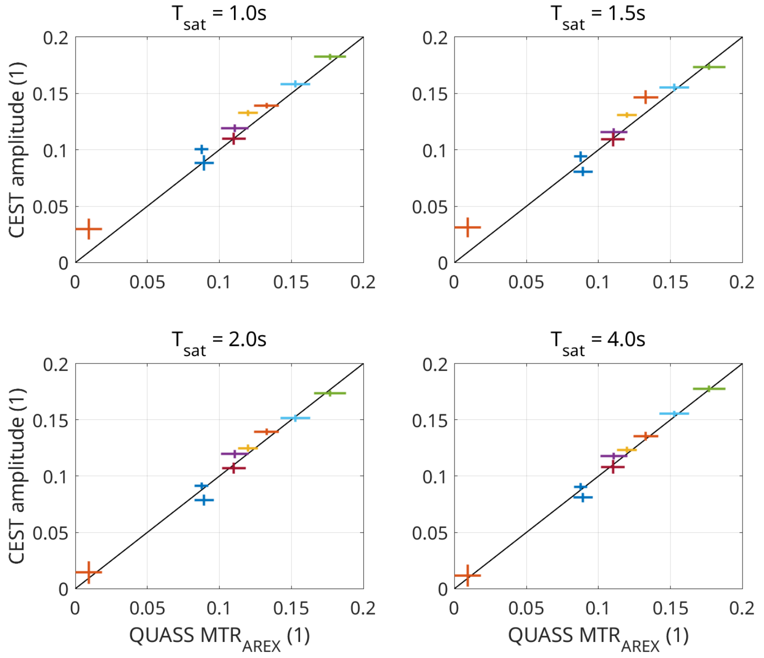

Figure 4: Means and standard deviations of all values inside the ROI shown in Figure 3 for all four saturation times. All comparisons are against the conventional CEST method with saturation time T_{sat}=4 s

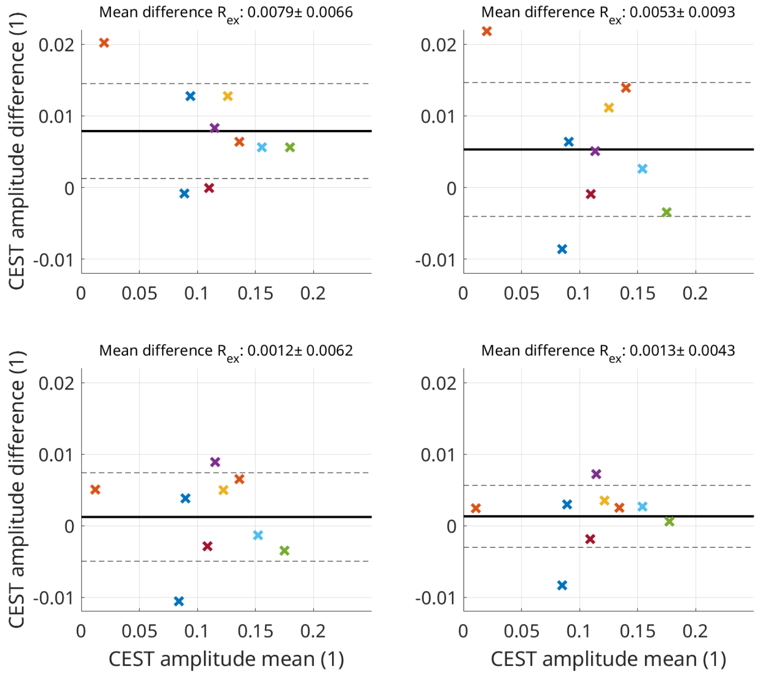

Figure 5: Bland Altman plots for all four saturation times. All comparisons are against the conventional CEST method with saturation time T_{sat}=4 s

Document S3 - Test-Restest experiment for the phantom measurement

The same phantom and sequences as described in the main text for the phantom measurement experiment were used. The proposed method, the reference IR-LL measurement, the reference QUASS MTR_{AREX} measurement and the WASABI measurement were each performed twice. After the first measurement, the scanner table was removed from the scanner and repositioned before the second measurement. Reconstruction and pre- and post-processing were done as described in the main text. The resulting maps of the second measurement were then registered to the first measurement using affine transformation implemented in BART. Maps of the T_1 values for the IR-LL and the

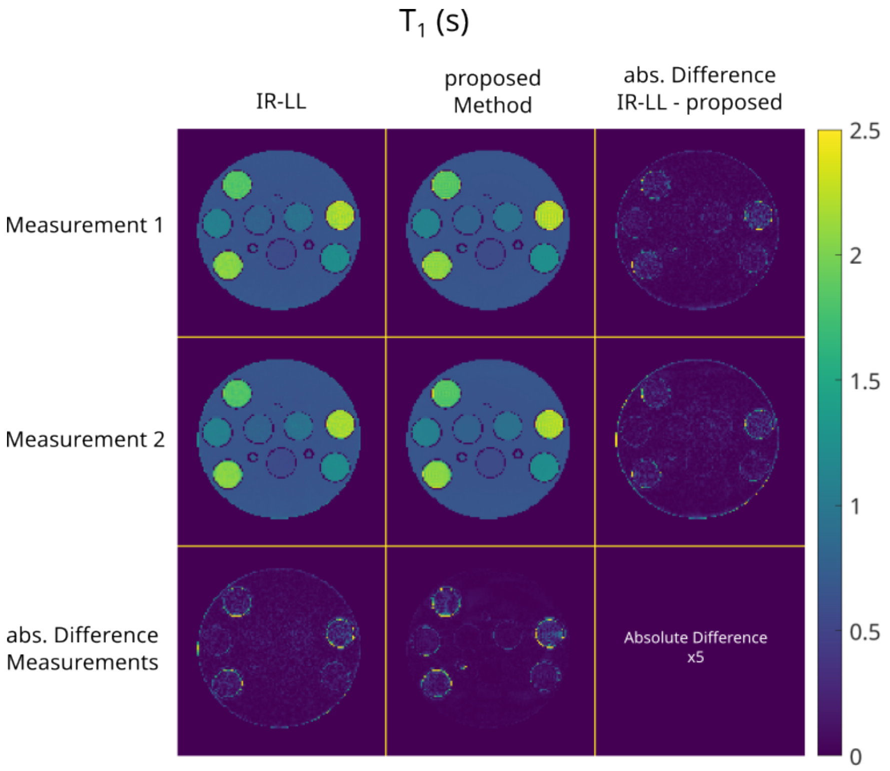

Figure 6: Resulting T_1 maps from the two measurements, for the reference IR-LL measurement and proposed method. Difference maps between the methods are shown in the right most column. Difference maps between the two measurements are shown in the bottom row. All difference maps are the absolute difference scaled by a factor of 5 for better visibility.

proposed method are shown in 6 together with the differences between the two measurements and the two methods. Maps of the CEST amplitude for the QUASS MTR_{AREX} and R_{ex} from the proposed method are shown in 7 together with the differences between the two measurements and the two methods. Mean and standard deviation of all ROIs for both measurements are shown in 8 for the T_1 values and in 9 for the CEST amplitude. The results show good repeatability for both the T_1 values and the CEST amplitude- The proposed methods shows similar repeatability as the reference methods. In the CEST amplitudes the conventional method shows more visible noise in

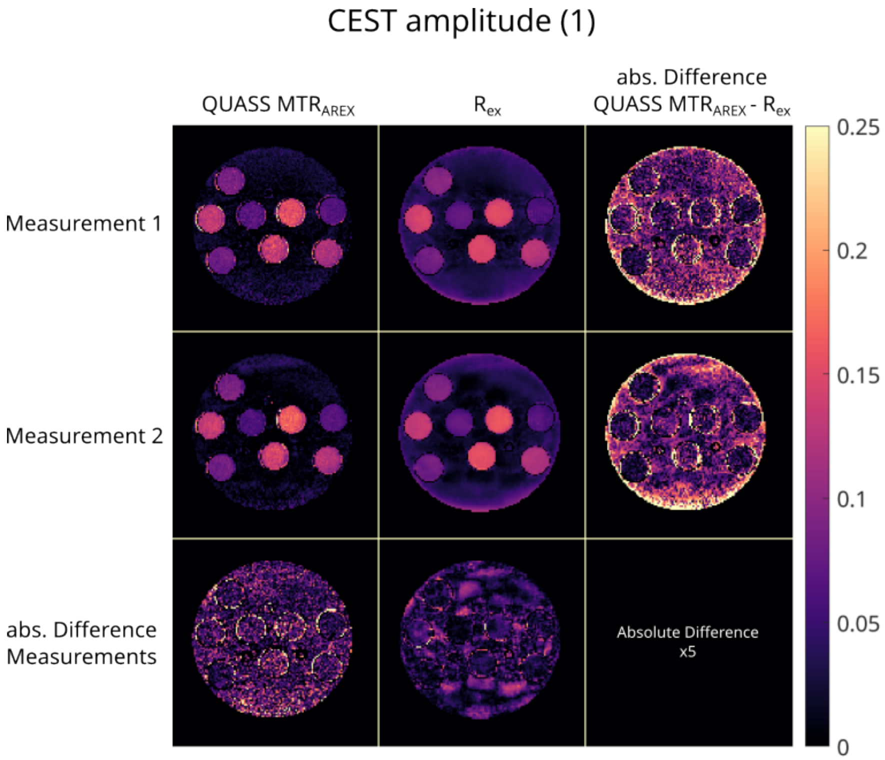

Figure 7: Resulting CEST amplitude maps from the two measurements, for the reference QUASS MTR_{AREX} measurement and R_{ex} from the proposed method. Difference maps between the methods are shown in the right most column. Difference maps between the two measurements are shown in the bottom row. All difference maps are the absolute difference scaled by a factor of 5 for better visibility.

the difference, while the proposed method shows a more uniform difference. This is expected as the model-based reconstruction uses spatial-TGV regularization, while the denoising in the reference method is done pixel-wise.

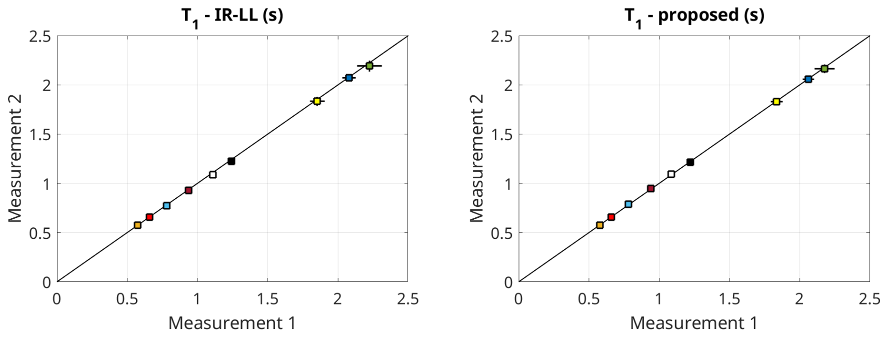

Figure 8: Mean and standard deviation for all ROIs for both measurements of the reference IR-LL measurement (left) and the proposed method (right).

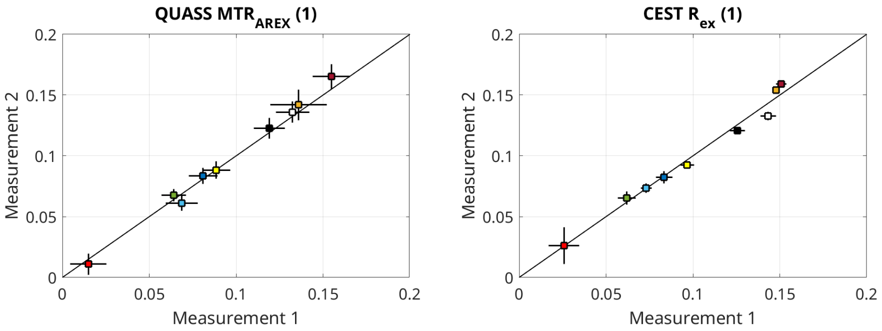

Figure 9: Mean and standard deviation for all ROIs for both measurements of the QUASS MTR_{AREX} measurement (left) and R_{ex} from the proposed method (right).

Document S4 - Further investigation of differences between QUASS MTR$_{AREX}$ and R_{ex}

In order to further investigate the differences between the QUASS MTR_{AREX} and R_{ex} from the proposed method, the differences were plotted against the B_0 inhomogeneity and relative B_1 for the center slice of in-vivo measurement. B_0 and B_1 maps are shown in Supplementary Figure S1 and the CEST maps in Figure 8 of the main text. The results are shown in Figure 10 and 11. With R^2 of 0.058 and 0.0271 only a weak correlation can be observed for both B_0 and B_1, indicating that the differences are not due to B_0 or B_1 inhomogeneities.

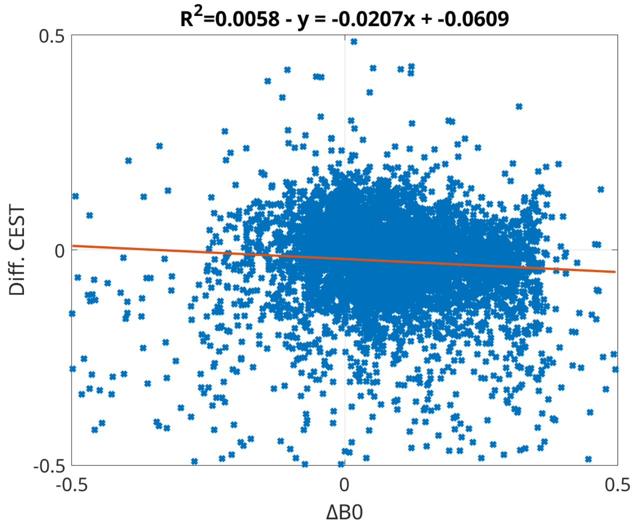

Figure 10: Difference between the CEST amplitudes of the QUASS MTR_{AREX} and R_{ex} from the proposed method plotted against the B_0 inhomogeneity for the in-vivo measurement.

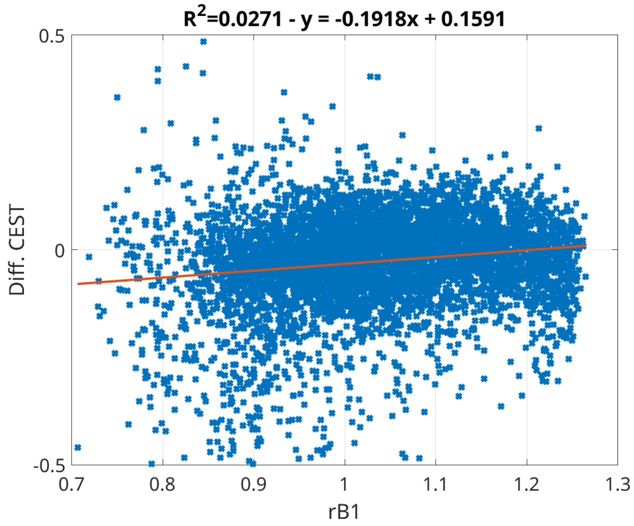

Figure 11: Difference between the CEST amplitudes of the QUASS MTR_{AREX} and R_{ex} from the proposed method plotted against the relative B_1 for the in-vivo measurement.

Figure S1 - B_0 and B_1 field maps of the in-vivo measurement

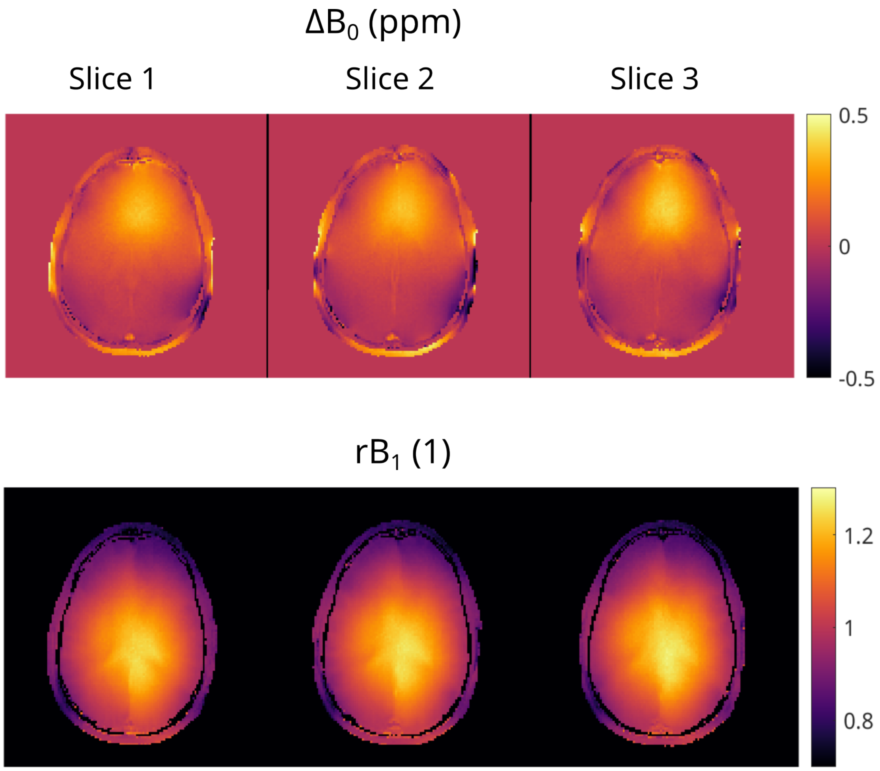

Figure S1: ΔB_0 and relative B_1 maps for the measured in-vivo slices

Table S1 - Optimization and regularization parameters for all experiments

Table S1: Regularization Parameters used in model-based reconstruction

Experiment	Model Name	γ_{start}	γ_{min}	reduction factor	Gauss-Newton steps
numerical phantom image-space	CEST T1 2 pool	1e-4	1e-10	0.7	15
numerical phantom k-space	CEST T1 2 pool	5e-2	1e-3	0.7	20
phantom measurement	CEST T1 2 pool	1e-1	1e-3	0.7	20
in-vivo measurement	CEST T1 4 pool	1e-2	1e-3	0.7	20
phantom and invo T_1 reference	IR-LL	1e-2	1e-3	0.5	10
